# Physiological Markers of Cybersickness in Virtual Reality: Skin Conductance Correlates with Momentary Cybersickness Across Time

**DOI:** 10.1177/29941520251415449

**Published:** 2026-02-20

**Authors:** Phillip Brown, Andrew T. Hendrickson, Pieter Spronck, Wendy Powell

**Affiliations:** Tilburg University, Tilburg, Netherlands.

**Keywords:** virtual reality, cybersickness, self-report, heart rate, electrodermal activity

## Abstract

This exploratory study investigates the feasibility of using physiological measures to assess cybersickness during virtual reality (VR) immersion, with a particular focus on within-subject correlations to explore the relationship between the cybersickness self-report and physiological measures. The cybersickness-inducing simulation showed a significant increase in cybersickness self-report, a significant increase in electrodermal activity (EDA) measures, skin conductance level (SCL), and skin conductance response (SCR), although no such difference existed for heart rate beats per minute (BPM). Importantly, a repeated measures correlation analysis revealed a significant positive within-individual relationship between self-reported cybersickness and EDA measures (SCL and SCR). These findings suggest that EDA measures may serve as a valuable continuous physiological measure of cybersickness during VR immersion.

## Introduction 

Cybersickness is a form of motion sickness often experienced during virtual reality (VR) exposure with a symptom profile that includes headache, nausea, vertigo, and dizziness.^1,2^ The prominent theory as for why cybersickness occurs is the sensory mismatch theory^2^ which proposes that cybersickness occurs due to vestibular and proprioceptive mismatch with visual cues.^3^ In a VR context, a virtual environment may trigger visual movement cues (vection), which may indicate that physical movement is currently happening, or rather the illusion of self-motion.^4^ In the real world, it is likely that we are not moving with the same velocity or in the same manner as the virtual environment depicts. In this case, the proprioceptive and vestibular cues indicate that the user may be stationary or moving in a different way, which in turn causes a sensory mismatch to occur and can trigger the onset of cybersickness symptoms.^5^

Aside from undesirable side effects, cybersickness has also been identified as negatively correlating with presence.^6^ Presence is the ability of VR to make users’ feel a sense of being present in a virtual environment.^7,8^ While this is crucial for VR capabilities as an entertainment medium, the ability to make users’ feel a sense of presence could have a profound impact on treatment or operational outcomes. One such example may be VR’s use as a pain distraction technique, as the basis of VR’s efficacy in this capacity is reliant users’ feeling a sense of presence, which in turn directs the users’ attention away from the painful experience and toward something more engaging and rewarding (i.e., the virtual environment).^9–11^

The most common measurement of cybersickness is the Simulator Sickness Questionnaire (SSQ).^12^ The SSQ is typically employed (and was originally intended) as a post-assessment questionnaire,^12^ although it has been highlighted more recently as also being of value when measured before the assessment.^13,14^ While it would be informative to measure cybersickness during VR immersion, primarily due to the number of questions in the SSQ, administering it during VR immersion would require a long break and likely cause breaks in presence, which are undesirable.^15^ This may be particularly relevant in situations where presence is integral, for example, during health care interventions.^9,16,17^

Alternative self-report methods for measuring cybersickness have been developed, such as the Fast Motion Sickness Scale (FMS),^18^ which is a single question 0–20 Likert scale assessment. Although single-question assessment is more suitable for during immersion assessment, issues due to breaks in presence may still arise. As such, physiological methods, including heart rate (HR) and electrodermal activity (EDA), have been explored as alternatives to cybersickness self-report assessment.^1,19–23^

HR as an assessment for cybersickness has often reported increases in average beats-per-minute (BPM) when compared to baseline measures or screen-based control condition,^1,19,24^ although this has not been unanimously observed.^20^ Work that has correlated post-intervention cybersickness assessment and BPM indicates that a positive relationship may exist.^25^

Regarding components of EDA and its relation to cybersickness, a greater degree of disagreement exists. There majority of evidence indicates an increase in skin conductance levels (SCL)—the “tonic” slow moving component—when experiencing a condition that may cause cybersickness,^1,23^ although nonsignificant differences have also been observed.^26^ Limited work exists exploring the relationship between cybersickness and “phasic” EDA components, particularly skin conductance responses (SCR), which is the component of the signal usually represented by the number of “peaks” that measures quick changes in arousal often related to an event or activity. Gavgani et al.,^1^ found significant increases in SCR (forehead located) when comparing the last minute of their cybersickness intervention to baseline, although no significance was found at the first minute of their intervention or when reporting via finger skin conductance sensors.

The vast majority of previous studies that have focused on the prediction of cybersickness have done so with measures of cybersickness collected after the VR experience.^25,27^ While these approaches have enabled classification and prediction using machine learning models, they inherently treat cybersickness as a static, aggregated outcome. In contrast, our study introduces a momentary assessment framework by measuring self-reported cybersickness at regular intervals during the immersive experience, as well as continuous physiological measurement. This approach enables us to investigate the temporal dynamics of cybersickness and test the within-subject relationship between physiological assessment (e.g., HR, skin conductance) and subjective discomfort over time. We argue that this shift from a static to dynamic assessment offers a more ecologically valid and theoretically grounded view of how cybersickness emerges and fluctuates—a methodological leap that may inform both adaptive VR systems and future predictive modelling efforts.^28^

### Summary and aims

The usefulness of identifying cybersickness symptomology using alternatives to self-report methods is becoming more apparent, and being able to measure this during VR immersion would be beneficial. In addition, it would be informative to better understand the relationship between physiological and self-report measures of cybersickness.

Therefore, the aim of this work was to identify which physiological markers were indicative of cybersickness as well as whether there were potential relationships between cybersickness and physiological responses. The self-report measures and physiological measures were evaluated to see if they significantly changed throughout the intervention. In addition, the within-person relationship between self-report and each individual physiological measure was evaluated using a repeated measures correlation analysis^29^—a correlation analysis that controls for individual differences.^30^ As far as we are aware, this is the first study to examine the possibility of using physiological signals as a measure of changes in cybersickness within an individual.

## Methods

### Participants

The study was conducted with 20 healthy participants. Most participants had some VR experience, although this was generally minimal (e.g., a single previous exposure). Refer to Supplementary Appendix S1 for participants demographical information, including participants’ previous VR experience.

Participants were recruited via the Tilburg University participant pool. Participant inclusion criteria included being 18 years or above, being able to read and understand the information sheet and questionnaires in English, having no sight or hearing impairments (not correctable with hearing aids or lenses), no heart, circulatory, or blood pressure problems, or medical conditions such as diabetes, or epilepsy. The study was ethically reviewed by the Tilburg University advisory board (TSHD_RP50_56-60-2022).

### Materials

#### Self-report questionnaires

##### Fast Motion Sickness Scale

The FMS^18^ is a single-question verbal scale commonly used to assess motion sickness and is scored on a scale of 0–20, with 0 being no sickness at all to 20 being severe sickness. This was administered before, during, and after the cybersickness intervention.

##### Simulator Sickness Questionnaire

The SSQ^12^ is a 16-item questionnaire that asks participants on a scale of 0–3 how much of a particular cybersickness-associated symptom they are experiencing at a given moment. This was administered before and after the cybersickness intervention. Scoring for the SSQ was performed using an unweighted approach,^31^ with the total score the difference of post minus pre to alleviate potential pre-exposure symptom bias.^13^

### Hardware

#### VIVE Pro Eye

The VIVE Pro Eye^32^ VR Head Mounted Display (HMD) was used to present the VR scenario. It has a 110-degree field of view, a combined resolution of 2880 × 1600, and a refresh rate of 90 Hz.

#### Empatica E4 wristband

The Empatica E4^33^ is a noninvasive wearable device capable of recording HR via Photoplethysmogram (PPG) (sample rate—1 Hz) and skin conductance via EDA with a sample rate of 4 Hz.

#### EmTeq Pro

The EmTeq Pro^34^ is a multi-sensor wearable face mask that is inserted into an HMD headset, comprising of a 7-contact f-EMG sensor, a PPG sensor, and 3-axis IMU. All sensors are sampled at 1000 Hz.

### Software

**FIG. 1.**
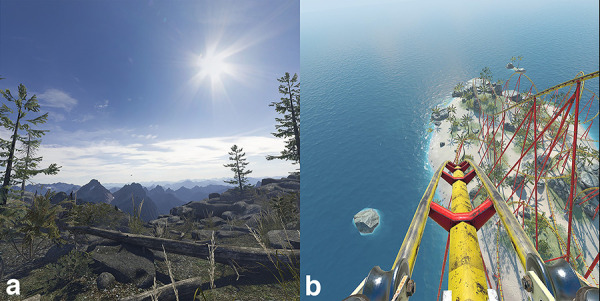
(**a**, Left) Screenshot of SteamVR Home (Valve, 2016) used for the control phase. (**b**, Right) Screenshot of Epic Roller Coasters (Games, 2018) used for the cybersickness phase.

Epic Roller Coasters^35^ (Fig. 1b) is commercial off-the-shelf software that simulates a roller coaster in VR. The user remains seated while they experience a roller coaster simulation, which we intended to cause a sensation of cybersickness. This did not require participant interaction to control. To further prompt onset cybersickness symptoms, we toggled the settings to disengage user interface elements intended to prevent cybersickness symptoms.

### Study design and procedure

Participants were welcomed and given time to ask questions about their participation before consent was obtained. Participants were made explicitly aware that they were able to withdraw at any time, without having to give a reason. The study was conducted in an air-conditioned room, which was kept at a constant 20°C. Half of the participants recruited were randomly assigned to this study, and the other half were assigned to a separate study intended to induce pain. Only data from the cybersickness induction study are included here.

Participants were fitted with an Empatica E4 wristband^33^ to their dominant arm, used to collect HR BPM and EDA data, and the participant rested for 10 min so that the physiological signals could drop to resting levels. Participants then answered a series of pre-intervention self-reports, which included the FMS and SSQ for cybersickness assessment. All participants were then fitted with an HTC Vive Pro eye HMD,^32^ which was modified with an EmTeq Pro face mask.^34^ The EmTeq Pro is fitted with a secondary PPG sensor, which was used for measuring BPM. Participants were guided through a calibration session for this.

Participants then partook in a 5-min control phase, which would require them to remain still while immersed in a passive virtual environment (Fig. 1a). After this period, participants asked to complete the FMS. Participants would then be immersed in VR for a maximum of 5 min for the intervention and were asked to answer the FMS at 30 s intervals.

The intervention involved participants experiencing a roller coaster simulation; Epic Roller Coasters^35^ (Fig. 1b). Participants would remain seated for the intervention, in which they would be free to navigate the experience visually and rotationally. The intervention would not require the participant to interact otherwise. The duration of the intervention was 2.5-min, which was repeated twice for a 5-min intervention in total. The visual aids intended to minimize the symptoms of cybersickness were disabled. This was intended to induce cybersickness symptoms, but not so severe that participants would quit the intervention, c.f. Gavgani et al.^1^At the end of the 5-min phase, the headset was removed. Post-intervention FMS and SSQ questionnaires were administered.

### Data processing and analysis.

All analyses between the EmTeq Pro facemask and the Empatica E4 wristband were synchronized for post-intervention analysis via Unix timestamps and events that were marked during the intervention.

**FIG. 2.**
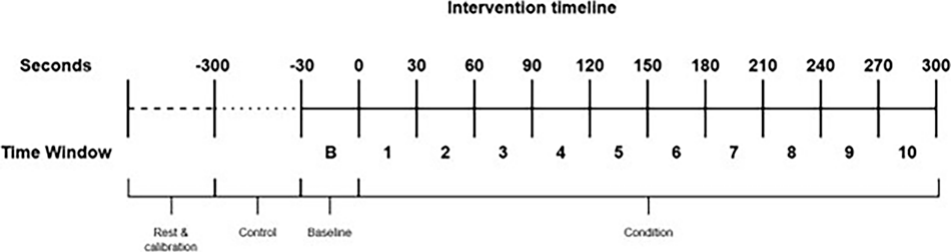
Timeline of intervention.

For the analysis of time-series data, the 5-min intervention period was split into 10 windows of 30 s each (Fig. 2). The last 30-s of the control intervention were used as a baseline measure.

### Physiological signals

HR was measured using the EmTeq Pro PPG (1000 Hz, downsampled to 25 Hz) as beats per minute (BPM).

Skin conductance was measured with the Empatica E4 GSR sensor. From this, we were able to extract the slow-changing tonic component, SCL, as well as the rapidly moving phasic component of the signal, SCR, from each 30 s time window. SCL was computed as the average skin conductance amplitude (µS). SCR was calculated by filtering the raw data and removing artifacts before counting the number of peaks. To achieve this, a bidirectional, first-order Butterworth filter using high pass (0.2 Hz) and low-pass (1 Hz) frequencies was used, followed by manual artifact removal. Peaks were defined with minimum amplitudes of 0.02 µS and a slope rate of <2 µS c.f.^36^

All physiological signals were baseline normalized. These corrected values were used in the correlation and slope analysis.

## Results

We first checked the assumptions of normality. This was done for each measure using the Shapiro-Wilk test of normality. Second, we evaluated the self-report scores to determine if the intervention was suitable at inducing cybersickness. Third, we checked to see if significant differences in the physiological measures existed between baseline and during the cybersickness phase. At last, we evaluated the correlations between momentary self-report and each physiological measure. A Holm-Bonferroni correction was used for all post-hoc comparisons that require a correction for multiple comparisons.

### Normality tests

Both the FMS and SSQ self-reports were not normally distributed. Regarding BPM, all time windows were normally distributed, except for time window 4. SCL and SCR were not normally distributed. As most of our measures were not normally distributed, nonparametric tests were performed in all cases for consistency.

### Self-report measures

#### Fast motion sickness scale

**FIG. 3.**
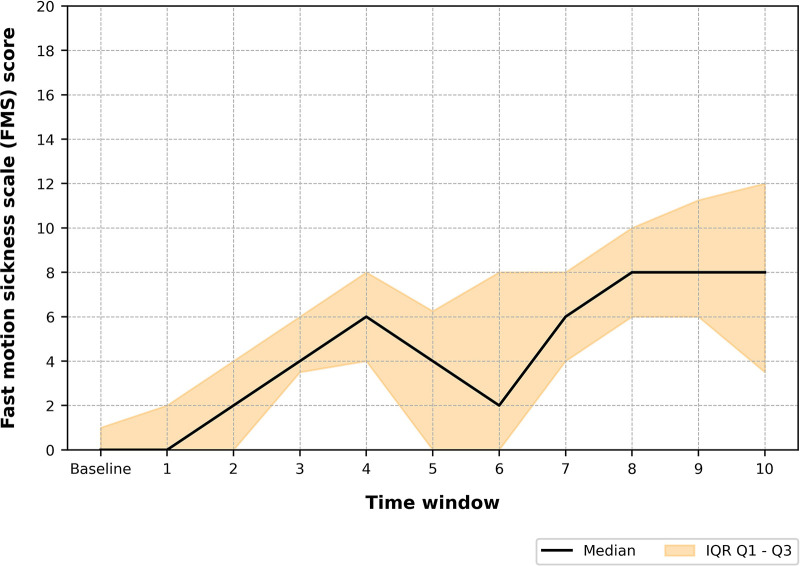
The distribution of self-reported cybersickness scores (FMS) in 30 second time windows throughout the cybersickness phase. Median line is marked, with Q1–Q3 Interquartile ranges surrounding. Note: The repeated nature of the cybersickness intervention (see Section 2.4) likely produced a decrease in self-reportedcybersickness during time windows 5 and 6.

A Friedman test shows a statistically significant difference in cybersickness self-report when comparing the 11 time windows (Fig. 2), χ^2^(10) = 98.893, *p* < 0.000. Planned post-hoc Wilcoxon signed-rank tests comparing between baseline and each other time window indicated there was a significant increase in reported cybersickness at 6 of 10 time windows (Fig. 3). See Supplementary Appendix S2 for full results.

#### Simulator sickness questionnaire

**FIG. 4.**
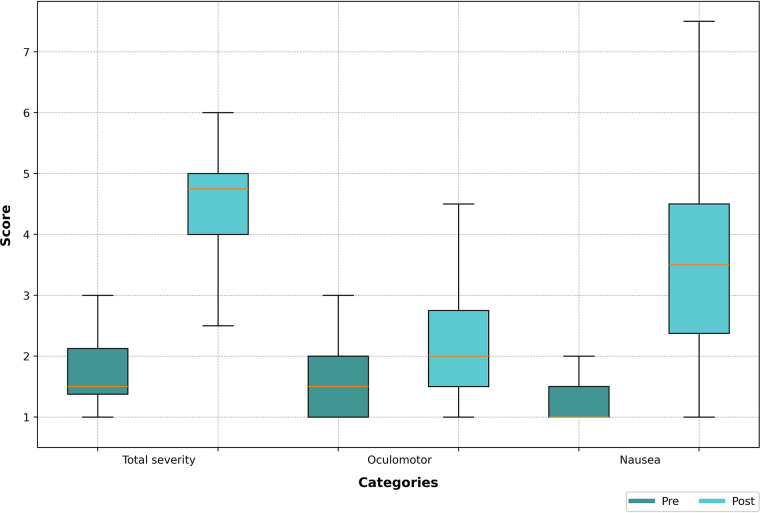
The distribution of Simulator Sickness Questionnaire (SSQ) scores before (pre) and after (post) the cybersickness intervention. Scores for the total score and each sub-scale score are displayed. Median line is marked, with boxes indicating Q1–Q3 Interquartile range. Whiskers indicate Min–Max values.

A Wilcoxon signed-rank test indicated that a significantly greater total SSQ score was observed post cybersickness intervention compared to pre-intervention assessment (z = −3.613, *p* < 0.000) (Fig. 4). See Supplementary Appendix S3 for a breakdown of subscale scores.

### Physiological measures

#### Beats per minute

**FIG. 5.**
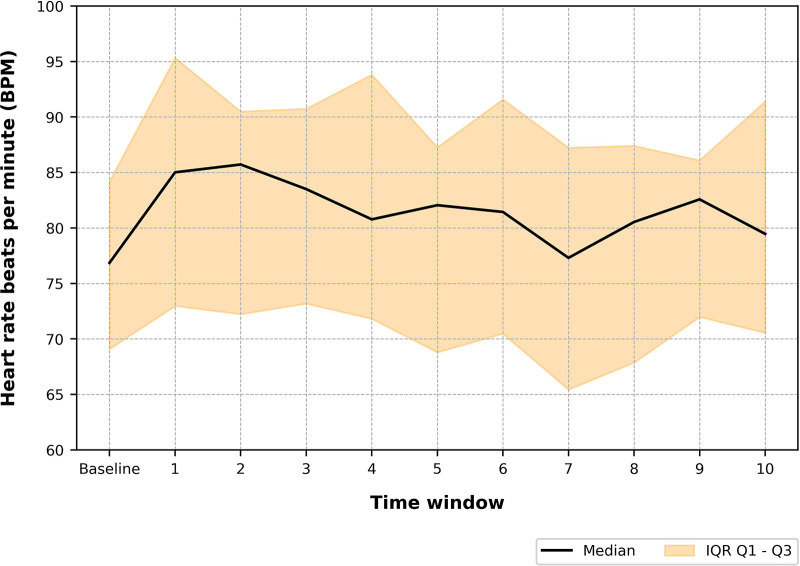
The distribution of Heart Rate Beats Per Minute (BPM) in 30 second time windows throughout the cybersickness phase. Median line is marked, with Q1–Q3 Interquartile ranges surrounding.

To determine if there were any significant differences in BPM across the 11 time windows (Fig. 2), a Friedman test was conducted. The results do not show a significant difference across time, χ^2^(10) = 18.053, *p* = 0.54. The planned post-hoc Wilcoxon signed-rank comparisons were conducted and are reported in Supplementary Appendix S4. These tests did show a significant difference for some time windows (Fig. 5).

#### Skin conductance levels

To determine if there were any significant differences in SCL across the 11 time windows (Fig. 2), a Friedman test was conducted. The results show there is a significant difference does exist, χ^2^(10) = 51.506, *p* < 0.000.

**FIG. 6.**
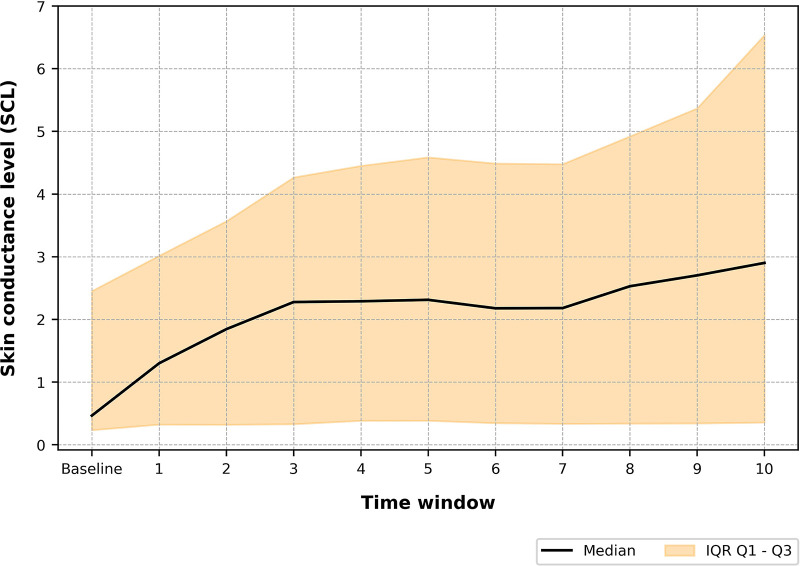
The distribution of Skin Conductance Level (SCL) measured in microsiemens (μS) in 30 second time windows throughout the cybersickness phase Median line is marked, with Q1–Q3 Interquartile ranges surrounding.

A set of planned post-hoc Wilcoxon signed-rank tests were conducted and are reported in Supplementary Appendix S4. These tests indicated that a significantly greater SCL was observed at all time windows when compared to baseline SCL (Fig. 6).

#### Skin conductance responses

To determine if there were any significant differences in SCR across the 11 time windows (Fig. 2), a Friedman test was conducted. The results do show a significant difference, χ^2^(10) = 28.833, *p* = 0.001.

**FIG. 7.**
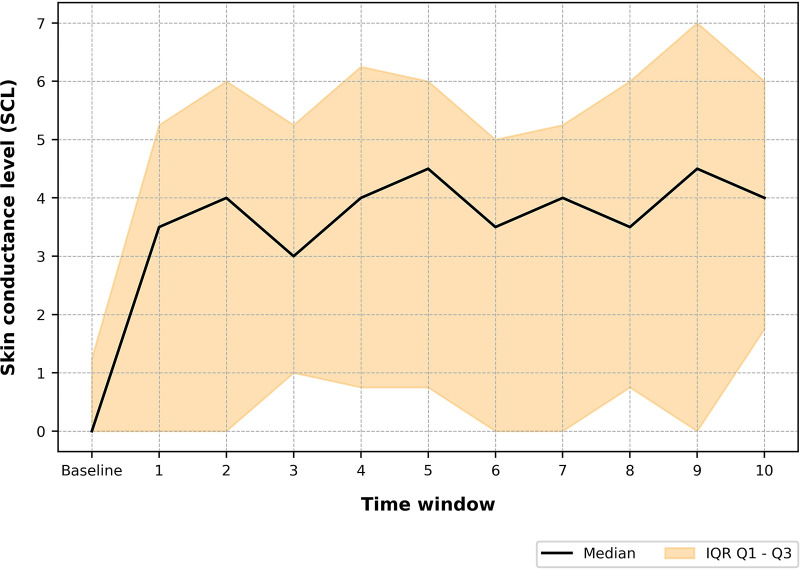
The distribution of Skin Conductance Response (SCR) measured in peaks in 30 second time windows throughout the cybersickness phase. Median line is marked, with Q1 – Q3 Interquartile ranges surrounding.

Planned post-hoc Wilcoxon signed-rank tests were conducted, also reported in Supplementary Appendix S4. Significantly greater SCR was observed at all time windows (Fig. 7).

### Correlation tests

Repeated measures correlations were run using the repeated measures correlation (rmcorr)^29^ package in R to determine whether a relationship existed between the physiological measures and self-reported cybersickness (FMS). For each individual, the physiological and FMS self-report were collected 11 times for the 30 s time windows (Bland & Altman, 1995). The repeated measures correlation analysis is similar to a multi-level model with an intercept for each individual but one slope that is shared across all individuals. The repeated measures aspect of this analysis increases statistical power compared to an inter-person correlation analysis while not requiring as much data as a full multi-level model, which estimates a full set of individual differences parameters.^30^

#### Beats per minute

**FIG. 8.**
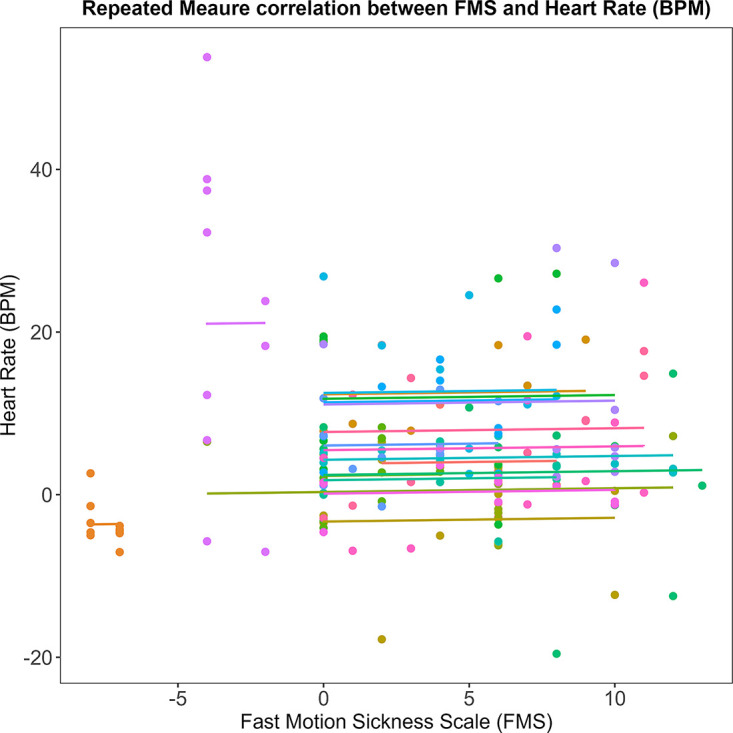
Repeated measures correlations between heart rate BPM and cybersickness self-report (FMS). Colours indicates individual participant ID.

There was not a significant inter-person correlation between BPM and cybersickness self-report (FMS) (r_rm_(157) = 0.02, 95% CI: [−0.138, 0.174], *p* = 0.818) (Fig. 8).

#### Skin conductance levels

**FIG. 9.**
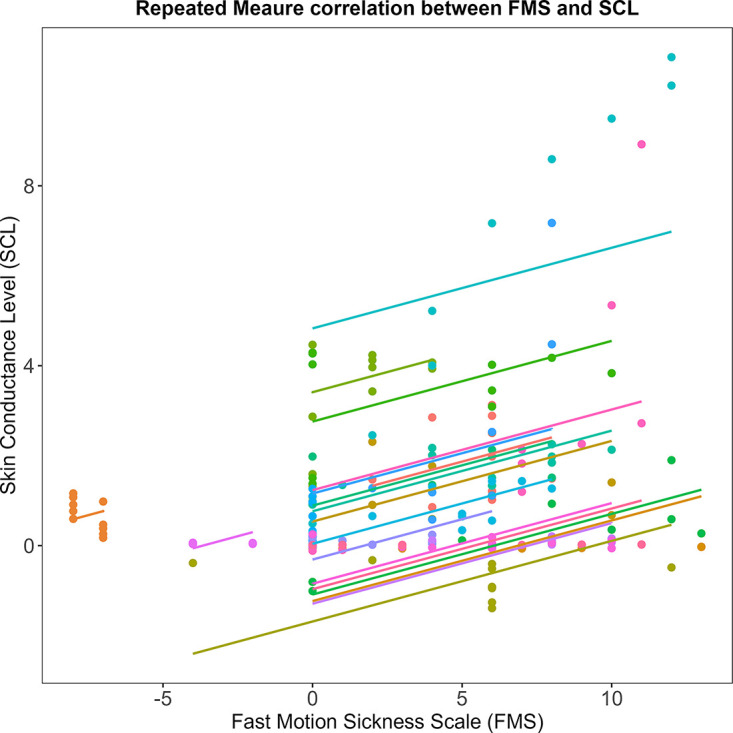
Repeated measures correlations between Skin Conductance Levels (SCL) and cybersickness self report (FMS). Colors indicates individual participant ID.

There was a significant positive correlation between SCL and cybersickness self-report (FMS) (r_rm_(179) = 0.45, 95% CI: [0.329, 0.562], *p* < 0.001) (Fig. 9).

#### Skin conductance response (SCR)

**FIG. 10.**
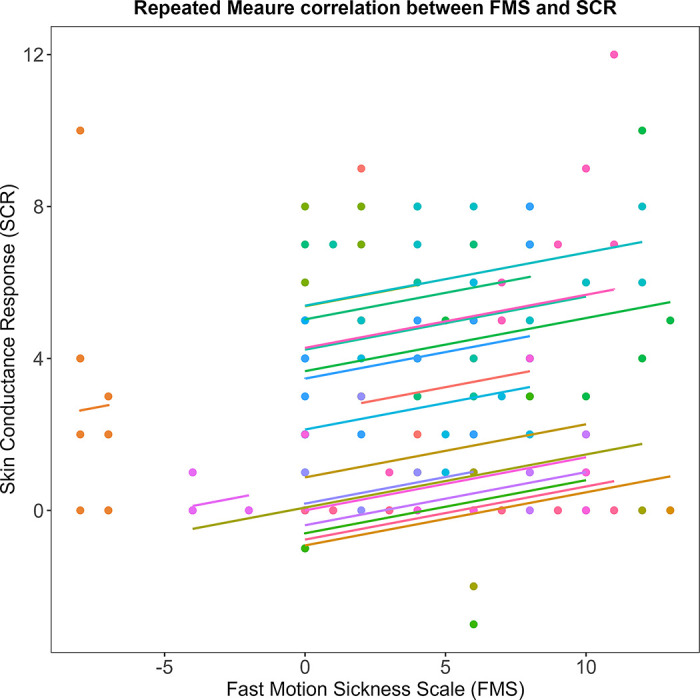
Repeated measures correlation between Skin Conductance Responses (SCR) and cybersickness self-report (FMS). Colors indicates individual participant ID.

As with SCL, there was a significant positive correlation between SCR and cybersickness self-report (FMS) (r_rm_(179) = 0.24, 95% CI: [0.095, 0.371], *p* = 0.001) (Fig. 10).

#### Individual slope analysis

To explore inter-individual variability and assess the robustness of the group-level associations, we calculated individual linear slops between baseline-corrected physiological (HR BPM, SCL, and SCR) and self-reported cybersickness (FMS).

For HR BPM, no reliable pattern was observed (*M* = −0.04 ± 0.47, range = −1.70 to 0.60), which is consistent with the non-significant group-level correlation (r_rm_ = 0.02). The SCL average slope was positive (M = 0.18 ± 3.04, range = −6.40 to 4.92), indicating that a substantial proportion of participants exhibited positive association between SCL and FMS (r_rm_ = 0.45). SCR showed a comparable trend (M = 0.26 ± 0.73, range = −0.75 to 1.87), which aligns well with our previous rmcorr result (r_rm_ = 0.24).

## Discussion

The aim of this exploratory study was to observe whether physiological methods may be suitable for measuring cybersickness during VR immersion and whether potential relationships may exist between the physiological and self-report measures. First, we evaluated the degree to which this simulation was successful in inducing cybersickness. We observed significantly higher post-assessment SSQ and FMS scores when compared to pre-assessment, suggesting cybersickness increased due to the simulation, and the FMS aligned with the SSQ results. In addition, when comparing pre-intervention FMS scores to each of the time windows throughout the condition, 6 of the 10 time windows reported a significant difference. The only time windows not reporting a significant difference corresponded to less intense moments of the virtual environment (i.e., when slowly climbing a hill in the roller coaster). At these moments, it would therefore be expected that the severity of symptoms may be less. Furthermore, the setup of the study (seated, on-rails movement, minimal participant control), the screening for related fear-associated disturbances, and the momentary cybersickness assessment throughout give us particular confidence in the validity of the simulation inducing varied cybersickness severity throughout.

Concerning HR BPM, we somewhat surprisingly observed no significant increase between the total intervention average and baseline average. This contrasts with previous literature indicating elevated BPM in cybersickness induction simulations compared to baseline.^1,19,24,37^ The absence of significant differences may be attributed to shorter exposure times in our study compared to those reporting significant increases.^20^ Planned post-hoc comparisons of individual time windows (reported in Supplementary Appendix S4) suggest there may have been some portions of the simulation with elevated BPM. This may indicate that further exploration of exposure length and relying less on total intervention averages to infer change to detect meaningful changes.

Regarding components of EDA, we reported that significant differences existed as an average of the total intervention for both SCL and SCR when compared to baseline. This broadly aligns with existing literature^1,23,25,37^ though see.^26^ We also found that for both components, each time window during the intervention compared to baseline was significantly greater. Few studies to date have explored the use of EDA SCR to infer cybersickness reaction, and of the work that has our results broadly align.^1,23,24^

An exciting finding from our analyses are the reliable, significant within-individuals correlation between self-reported cybersickness and some physiological measures. In particular, we found significant positive correlations with SCL and SCR but not with BPM (see *Correlation tests* section). Although correlation coefficients for both SCL and SCR are moderate in strength, both are significant and match those found in previous research.^1,20,37^This suggests that either SCL or SCR may be used as a nonintrusive cybersickness measurement technique. In addition, these correlations support that the FMS may be a reliable indicator of cybersickness during an intervention where EDA measurement may not be feasible.

An interesting point of attention is the contrast between the variability in individual slopes and the high confidence on the estimate of correlations between SCL and FMS. The ability to accurately estimate the correlation across individuals is a strength of the repeated measures correlation analysis that takes advantage of pooling across participants. Most notably, the narrow confidence intervals that do not include zero suggest an accurate measurement of the relationship between SCL and FMS.^29^ However, the variability in estimated slope across individuals does raise concerns about directly using SCL as the only measure of momentary cybersickness.

Despite these concerns, these are the first results to demonstrate this moment-to-moment relationship and suggests productive future research directions. One possibility is using a combination of SCL and SCR, or additional measures, to more accurately infer perceived cybersickness. Despite both being derived from EDA data, future research can clarify whether they capture unique variations in cybersickness or whether they could be combined together to capture more variation than either measure alone.

The lack of significant correlation between BPM and subjective cybersickness contrasts with prior research suggesting moderate relationships (c.f. Islam et al.^25^). This highlights potential limitations that BPM may not be a reliable indicator of cybersickness, especially during shorter VR exposures, warranting further investigation within varied durations and simulation intensities.

While we did screen participants for fear of heights and roller coasters, we cannot rule out the possibility that this may still confound some of our findings, as the literature does suggest similar physiological profiles to a potential cybersickness response.^38,39^ Similarly, confounders may exist for physiological assessment of anxiety, which we did not measure in this study. Future studies may look to confirm these findings, which account for the aforementioned conditions.

Furthermore, while the purpose of this article was not to make inferences about how cybersickness may be predicted via physiological methods, we do propose that a linear relationship between certain physiological and self-report methods may exist. By capturing cybersickness continuously throughout the VR experience, this study provides a more granular understanding of how symptoms evolve in real time—an approach that may offer valuable insights. Momentary increases in self-reported cybersickness, especially when aligned with shifts in physiological signals, could reflect disruptions in the user’s sense of immersion or coherence within the virtual environment. Importantly, this temporal resolution in measurement provides a rich foundation for the future development of predictive models.

Although in-immersion assessment of cybersickness was possible due to the use of proprietary software for cybersickness induction, future research could explore alternative methods that allow for continuous assessment during immersion e.g.,^40,41^ Such approaches may help reduce potential breaks in presence by minimizing interruptions to the immersive experience.

Additionally, by incorporating momentary cybersickness measures, machine learning systems could be trained to anticipate the onset of cybersickness, enabling real-time adaptations in VR content to maintain comfort and avoid breaks in presence. In this way, the current work lays conceptual and methodological groundwork for predictive and adaptive VR systems that go beyond static, post-hoc assessments.

## Conclusion

The aim of the study was to investigate the feasibility of using physiological measures of cybersickness during VR immersion whilst exploring potential relationships between self-report and physiological measures. Critically, we found a significant within-individual relationship across time windows between self-reported cybersickness and two measures of EDA. We found a significant increase in cybersickness self-report, a significant increase in EDA measures from the baseline, and significant positive correlations between self-reported cybersickness and the EDA measures.

This work contributes towards the understanding of the relationship between cybersickness self-report and physiological measures in the context of VR experiences. The identification of a potential continuous physiological measure of cybersickness is an exciting advance and highlights that EDA components may be suitable indicators of cybersickness during VR immersion.

This work extends existing approaches to cybersickness assessment by providing evidence that momentary self-report and physiological measurement are dynamically related to during VR exposure rather than only at aggregated endpoints. Our findings support the feasibility of using repeated, within-session measurements to capture temporal unfolding of cybersickness, offering a richer and more actionable understanding of user experience. This methodological advance lays the groundwork for adaptive VR systems and for future machine learning approaches that integrate both subjective and physiological feedback.

## Authors’ Contributions

Conceptualization: P.B. Data curation: P.B. Formal analysis: P.B., A.T.H., P.S., and W.P. Investigation: P.B. Methodology: P.B., A.T.H., P.S., and W.P. Project administration: P.B., A.T.H., P.S., and W.P. Resources: P.B. Supervision: P.B., A.T.H., P.S., and W.P. Writing—original draft: P.B. Writing—review and editing: P.B., A.T.H., P.S., and W.P.

## Supplemental Material

sj-docx-1-mxr-10.1177_29941520251415449 — Supplemental material for Physiological Markers of Cybersickness in Virtual Reality: Skin Conductance Correlates with Momentary Cybersickness Across Time Supplemental material, sj-docx-1-mxr-10.1177_29941520251415449 for Physiological Markers of Cybersickness in Virtual Reality: Skin Conductance Correlates with Momentary Cybersickness Across Time by

sj-docx-2-mxr-10.1177_29941520251415449 — Supplemental material for Physiological Markers of Cybersickness in Virtual Reality: Skin Conductance Correlates with Momentary Cybersickness Across Time Supplemental material, sj-docx-2-mxr-10.1177_29941520251415449 for Physiological Markers of Cybersickness in Virtual Reality: Skin Conductance Correlates with Momentary Cybersickness Across Time by

sj-docx-3-mxr-10.1177_29941520251415449 — Supplemental material for Physiological Markers of Cybersickness in Virtual Reality: Skin Conductance Correlates with Momentary Cybersickness Across Time Supplemental material, sj-docx-3-mxr-10.1177_29941520251415449 for Physiological Markers of Cybersickness in Virtual Reality: Skin Conductance Correlates with Momentary Cybersickness Across Time by

sj-docx-4-mxr-10.1177_29941520251415449 — Supplemental material for Physiological Markers of Cybersickness in Virtual Reality: Skin Conductance Correlates with Momentary Cybersickness Across Time Supplemental material, sj-docx-4-mxr-10.1177_29941520251415449 for Physiological Markers of Cybersickness in Virtual Reality: Skin Conductance Correlates with Momentary Cybersickness Across Time by

sj-docx-5-mxr-10.1177_29941520251415449 — Supplemental material for Physiological Markers of Cybersickness in Virtual Reality: Skin Conductance Correlates with Momentary Cybersickness Across Time Supplemental material, sj-docx-5-mxr-10.1177_29941520251415449 for Physiological Markers of Cybersickness in Virtual Reality: Skin Conductance Correlates with Momentary Cybersickness Across Time by

## Abbreviations

BPM: Beats Per Minute
EDA: Electrodermal Activity
FMS: Fast Motion Sickness Scale
HMD: Head Mounted Display
HR: Heart Rate
rmcorr: repeated measures correlation
SCL: Skin Conductance Level
SCR: Skin Conductance Response
SSQ: Simulator Sickness Questionnaire
VR: Virtual Reality
