# Supplementary material for: Physiological Markers of Cybersickness in Virtual Reality: Skin Conductance Correlates with Momentary Cybersickness Across Time
Source: J Med Ext Real. 2026 Feb 20;3:29941520251415449. doi: 10.1177/29941520251415449 (PMC13383644; doi:10.1177/29941520251415449)
Supplement: Supplementary file 1 [file sj-docx-1-mxr-10.1177_29941520251415449.docx]

## Appendix A – Demographics

| Demographics | |
| --- | --- |
| **Age** | |
| 16 – 18 | 3 (15%) |
| 19 – 29 | 12 (60%) |
| 30 - 39 | 5 (25%) |
| **Sex** | |
| Female | 9 (45%) |
| Male | 11 (55%) |
| **Ethnicity** | |
| White | 18 (90%) |
| Mixed | 1 (5%) |
| Other/ Prefer not to say | 1 (5%) |
