# Supplementary material for: Physiological Markers of Cybersickness in Virtual Reality: Skin Conductance Correlates with Momentary Cybersickness Across Time
Source: J Med Ext Real. 2026 Feb 20;3:29941520251415449. doi: 10.1177/29941520251415449 (PMC13383644; doi:10.1177/29941520251415449)
Supplement: Supplementary file 2 [file sj-docx-2-mxr-10.1177_29941520251415449.docx]

## Appendix B – Within subjects comparisons, comparing self-report baseline to individual time windows

| Fast motion sickness scale (FMS) | | | | | | | | | | |
| --- | --- | --- | --- | --- | --- | --- | --- | --- | --- | --- |
| **Time Window** | **1** | **2** | **3** | **4** | **5** | **6** | **7** | **8** | **9** | **10** |
| Z | -1.289 | -1.832 | -2.710 | -3.173 | -2.334 | -2.282 | -3.109 | -3.376 | -3.410 | -3.209 |
| Sig | .197 | .067 | **.007** | **.002** | .020 | .023 | **.002** | **.001** | **.001** | **.001** |

Results of Wilcoxon signed-rank tests, comparing the self-report baseline and self-report at each time window. **Bold** indicates significance.
