# Supplementary material for: Physiological Markers of Cybersickness in Virtual Reality: Skin Conductance Correlates with Momentary Cybersickness Across Time
Source: J Med Ext Real. 2026 Feb 20;3:29941520251415449. doi: 10.1177/29941520251415449 (PMC13383644; doi:10.1177/29941520251415449)
Supplement: Supplementary file 3 [file sj-docx-3-mxr-10.1177_29941520251415449.docx]

## Appendix C – SSQ comparisons

| Simulator Sickness Questionnaire comparisons | | | |
| --- | --- | --- | --- |
|  | Total | Nausea | Disorientation |
| Z | -3.613 | -3.633 | -1.634 |
| Sig. | **.000** | **.000** | .102 |

Results of Wilcoxon signed-rank tests, comparing baseline SSQ total scores, and sub-scale nausea and disorientation scores. **Bold** indicates significance.
