# Supplementary material for: Physiological Markers of Cybersickness in Virtual Reality: Skin Conductance Correlates with Momentary Cybersickness Across Time
Source: J Med Ext Real. 2026 Feb 20;3:29941520251415449. doi: 10.1177/29941520251415449 (PMC13383644; doi:10.1177/29941520251415449)
Supplement: Supplementary file 4 [file sj-docx-4-mxr-10.1177_29941520251415449.docx]

## Appendix D - Within subjects comparisons, comparing physiological signals baseline to individual time windows

| Within subjects time series analysis of physiological signals | | | | | | | | | | |
| --- | --- | --- | --- | --- | --- | --- | --- | --- | --- | --- |
| **Time Window** | **1** | **2** | **3** | **4** | **5** | **6** | **7** | **8** | **9** | **10** |
| Beats per minute | | | | | | | | | | |
| Z | -2.817 | -2.391 | -2.012 | -2.391 | -1.823 | -1.491 | -1.917 | -2.722 | -2.485 | -2.343 |
| Sig. | **.005** | .017 | .044 | .017 | .068 | .136 | .055 | .006 | .013 | .019 |
| Beats per minute difference (%) | | | | | | | | | | |
| Z | -2.769 | -2.296 | -2.154 | -2.391 | -2.154 | -1.586 | -1.965 | -2.769 | -2.580 | -2.485 |
| Sig. | .006 | .022 | .031 | .017 | .031 | .113 | **.049** | .006 | .010 | .013 |
| Skin conductance level | | | | | | | | | | |
| Z | -2.315 | -2.464 | -2.987 | -3.211 | -3.323 | -3.285 | -3.360 | -3.360 | -3.397 | -3.584 |
| Sig. | **.021** | **.014** | **.003** | **.001** | **.001** | **.001** | **.001** | **.001** | **.001** | **< .0001** |
| Skin conductance level difference (%) | | | | | | | | | | |
| Z | -2.688 | -2.763 | -3.248 | -3.584 | -3.584 | -3.435 | -3.472 | -3.472 | -3.509 | -3.808 |
| Sig. | **.007** | **.006** | **.001** | **< .0001** | **< .0001** | **.001** | **.001** | **.001** | **.000** | **< .0001** |
| Skin conductance responses (peaks) | | | | | | | | | | |
| Z | -2.859 | -3.048 | -3.267 | -3.188 | -3.205 | -3.017 | -2.871 | -3.325 | -3.187 | -3.330 |
| Sig. | **.004** | **.002** | **.001** | **.001** | **.001** | **.003** | **.004** | **.001** | **.001** | **.001** |

Results of Wilcoxon signed-rank tests, comparing the baseline average and time window averages. **Bold** indicates significance.
