# Supplementary material for: Physiological Markers of Cybersickness in Virtual Reality: Skin Conductance Correlates with Momentary Cybersickness Across Time
Source: J Med Ext Real. 2026 Feb 20;3:29941520251415449. doi: 10.1177/29941520251415449 (PMC13383644; doi:10.1177/29941520251415449)
Supplement: Supplementary file 5 [file sj-docx-5-mxr-10.1177_29941520251415449.docx]

## Appendix E – Individual slope analysis summary

| Physiological individual signal slope analysis results | | | |
| --- | --- | --- | --- |
|  | BPM | SCL | SCR |
| **N** | 18 | 16 | 20 |
| **Mean (SD)** | -0.04 (0.47) | 0.18 (3.04) | 0.26 (0.73) |
| **Min - Max** | [-1.70, 0.60] | [-6.40, 4.92] | [-0.75, 1.87] |
| **25% – 75%** | [-0.10, 0.16] | [-1.05, 1.40] | [-0.23, 0.91] |

Results of individual slope analysis. Each slope was calculated between applicable physiological signals and self-reported cybersickness (FMS).
